# Robotic simultaneous resection of rectal cancer and liver metastases

**DOI:** 10.1002/ccr3.1138

**Published:** 2017-10-05

**Authors:** Supreet Sunil, Juliana Restrepo, Arash Azin, Dhruvin Hirpara, Sean Cleary, Michelle C. Cleghorn, Alice Wei, Fayez A. Quereshy

**Affiliations:** ^1^ Division of General Surgery University Health Network Toronto Ontario Canada; ^2^ Division of General Surgery University of Toronto Toronto Ontario Canada; ^3^ Faculty of Medicine University of Toronto Toronto Ontario Canada

**Keywords:** Colorectal cancer, liver metastases, malignancy, oncology, robotic surgery, synchronous liver metastases

## Abstract

Surgical resection is the only potential cure for colorectal cancer with synchronous liver metastases (SLM). Simultaneous resection of colorectal cancer and SLM using robotic‐assistance has been rarely reported. We demonstrate that robotic‐assisted simultaneous resection of colorectal cancer and SLMs is feasible, safe, and has potential to demonstrate good oncologic outcomes.

## Introduction

Colorectal cancer (CRC) is the fourth leading cause of cancer‐related death in the world 1. Up to 25% of newly diagnosed patients with CRC and 50% of those undergoing clinical management may present with synchronous metastasis, with the liver being the most common site for metastatic lesions 2. For patients with colorectal liver metastasis (CRLM), the treatment strategy should largely be directed toward resectability. Without resection, median overall survival (OS) for patients with CRLM ranges from 20 to 24 months with modern chemotherapeutic regimens 3. In those where an R_0_ resection of all metastatic disease is achieved, the 5‐year OS has been reported to be as high as 58% 2, 3, 4.

Patients with synchronous CRLM can be treated with three different strategies; a liver first approach, a staged approach, or a simultaneous resection. Several retrospective studies comparing these approaches have demonstrated that simultaneous hepatic and colorectal resections are safe in select patients 5, 6, 7, 8, 9, 10. Given the benefits of minimally invasive surgery compared to open surgery, there has been increased interest in feasibility of robotic‐assisted liver resection (RALR). Studies have demonstrated that outcomes following RALR are comparable to those of laparoscopic liver resections 11, 12, 13, 14. Although simultaneous laparoscopic colorectal and liver resections have been described, the literature is sparse with regard to simultaneous robotic‐assisted liver and colorectal resections. We report a case of a patient with colorectal cancer and synchronous liver metastasis that underwent successful simultaneous robotic‐assisted resection of a rectosigmoid cancer and colorectal liver metastasis.

## Case History/Examination/Investigations

**Figure 1:**
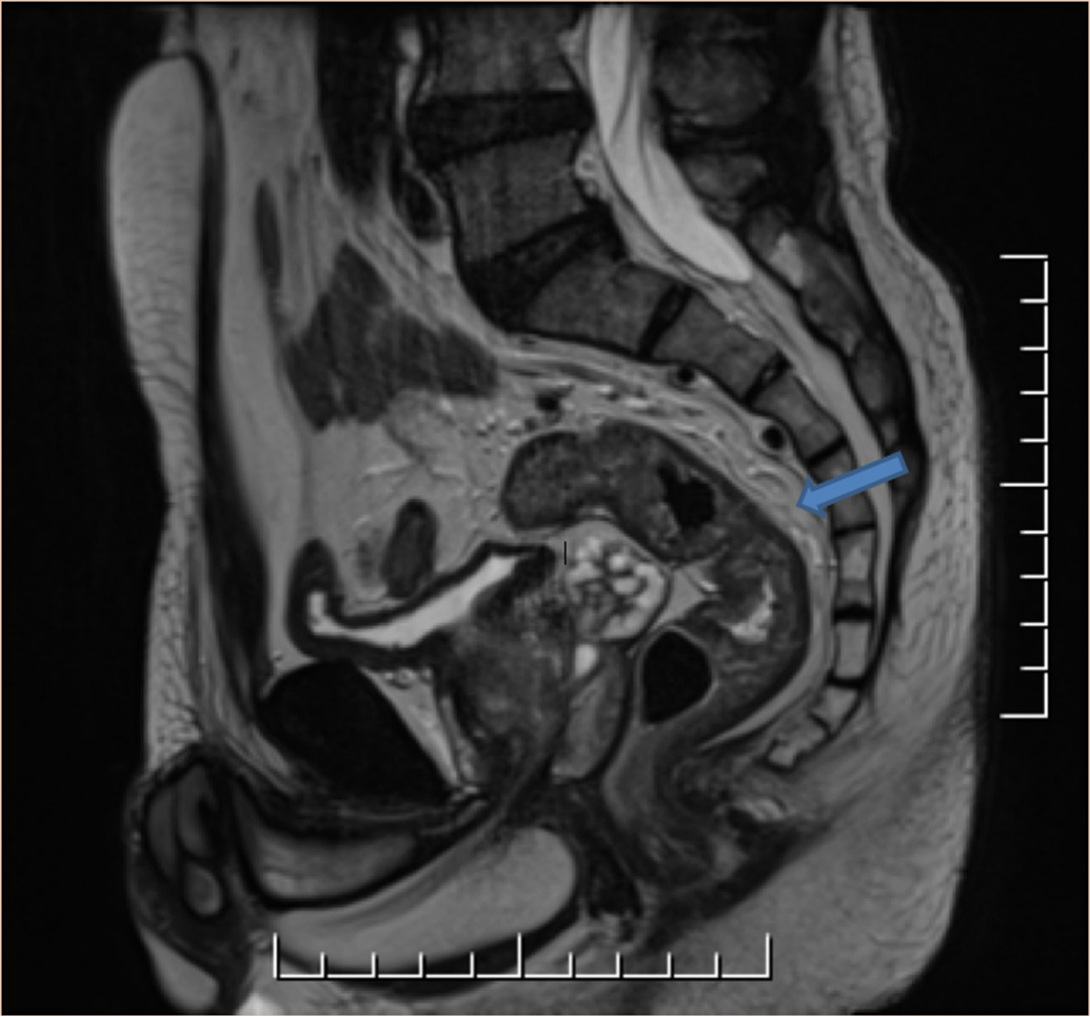
Sagittal MRI pelvis demonstrating T2/T3 rectosigmoid mass.

**Figure 2:**
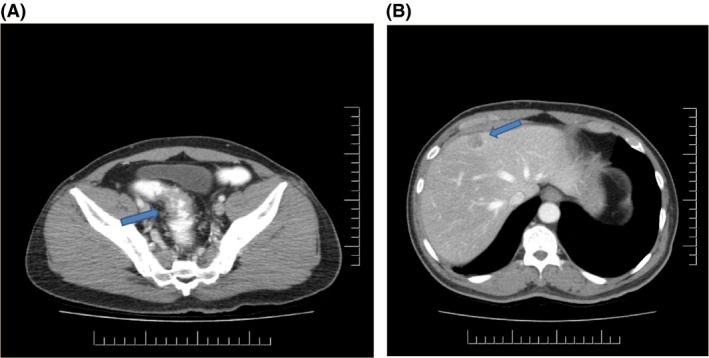
(A) Computerized axial tomography of pelvis demonstrating a 5‐cm mass at the rectosigmoid junction. (B) Computerized axial tomography of liver demonstrating a 2.1 cm suspicious lesion in segments 4a/8.

A 59‐year‐old man with a history of hypertension, diabetes, and dyslipidemia presented with occasional bright red blood per rectum and anemia. The patient did not endorse any personal or family history of malignancy, and denied all constitutional or dysenteric symptoms. Physical examination, including a digital rectal examination, was unremarkable. A subsequent diagnostic colonoscopy revealed a mass 12 cm from the anal verge, which was tattooed and biopsied. Histological examination revealed a moderately differentiated adenocarcinoma. Staging MRI demonstrated a T2 or possibly early T3 lesion above the peritoneal reflection with one indeterminate 5 mm lymph node within the sigmoid mesocolon (Fig. 1). CT scans of the chest, abdomen, and pelvis (Fig. 2) revealed a 5‐cm mass at the rectal sigmoid transition and a 2.1 cm lesion in segments 4a/8 of the liver concerning for metastases. A subsequent liver MRI, completed to fully work‐up three other lesions, confirmed a 1.8 cm metastatic deposit high over the dome of the diaphragm in segment 4a/8 of the liver and three smaller lesions that were likely hemangiomas and a cyst. This patient was discussed at multidisciplinary tumor boards where the consensus was that a simultaneous resection, without neoadjuvant therapy, would be feasible. Given the location of the metastatic liver deposit high over the dome of the diaphragm, a robotic resection was proposed as it allowed advantages over a laparoscopic approach in ensuring greater access, ergonomics, and dexterity. Informed consent was obtained, and the patient was scheduled for a robotic low anterior resection (LAR) and a nonanatomical robotic liver resection with diverting ileostomy.

## Treatment

**Figure 3:**
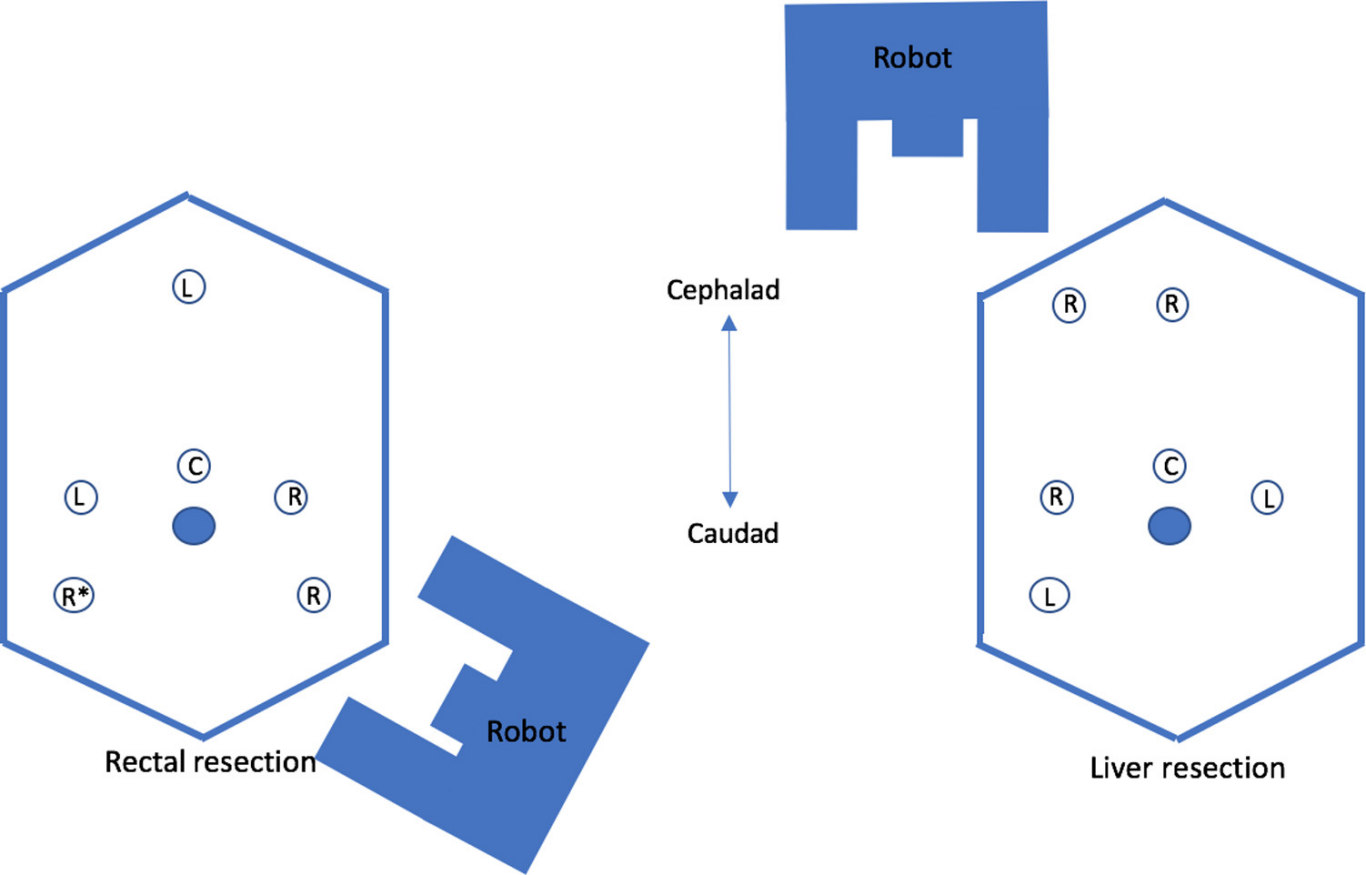
Schematic diagram of port placement for robotic rectal and liver resection. C – 12‐mm camera port, R – 8‐mm robotic port, R* ‐ 15‐mm robotic port, L‐ 5‐mm laparoscopic port.

The patient was appropriately positioned and the abdomen was entered using an open approach with placement of a 12‐mm supraumbilical camera port. A 15‐mm robotic trocar was placed in the right lower‐quadrant (RLQ) at the half‐way point between a line from the umbilical port to the anterior superior iliac spine (ASIS). Similarly, an 8‐mm robotic port was placed in the left lower‐quadrant (LLQ). A third 8‐mm robotic port was placed in the LLQ lateral and superior to the previous port. Two 5‐mm laparoscopic ports were subsequently inserted, one in the right midabdomen and one in the epigastric area (Fig. 3).

The da Vinci^®^ (Sunnyvale, CA, USA) surgical system model S (Intuitive Surgical, Sunnyvale, CA, USA) was docked on the inferior left side of the patient, with an approximate docking time of 15 min (Fig. 3). Mobilization of the left colon was conducted using a lateral to medial approach. Subsequently, the medial aspect of sigmoid colon was dissected, identifying the superior hemorrhoidal arch and superior mesenteric artery and vein (SMA/SMV). The IMA and IMV were skeletonized, and a regional lymphadenopathy was conducted. The superior hemorrhoidal branch was identified, cleared, and divided with surgical clips. A previously placed tattoo identifying the location of the tumor was identified at the level of the peritoneal reflection. A proximal resection margin was selected in the sigmoid colon 5 cm proximal to the tumor, and the mesentery was divided at this level using a 5‐mm LigaSure device. This was then followed by a total mesorectal excision (TME) to the level of the pelvic floor. A flexible sigmoidoscopy was then conducted to mark the location of our distal margin 5 cm from the tumor. At the location of our distal margin, the mesorectum was circumferentially divided to skeletonize the rectum. The rectum was divided using a linear laparoscopic GIA 60 purple tristapler to obtain a tumor‐specific TME (sTME). The da Vinci^®^ was undocked, with a total a total rectal dissection duration, including docking, of approximately 190 min. The specimen was exteriorized using a 5‐cm pfannenstiel incision and a wound protector device. The colon was divided approximately 5 cm proximal to the tumor, and the remaining colon was reintroduced into the abdomen after placement of a 28 EEA anvil in the bowel. Pneumoperitoneum was re‐established and a primary tension‐free laparoscopic colorectal anastomosis was fashioned using a 28 mm EEA circular stapler.

The epigastric and right mid abdomen 5‐mm ports were upsized to 8‐mm robotic ports. An additional 8‐mm robotic port was placed two fingerbreadths below the costal margin at the midclavicular line (Fig. 3). The robot was redocked over the patient's right shoulder, with an approximate docking time of 15 min. The metastatic liver lesion was identified high over the dome of the diaphragm at segment 4a/8 of the liver. An intraoperative liver ultrasound was conducted to rule out other liver lesions and to determine the transection line and resection margins of the aforementioned lesion. No additional malignant lesions were identified by an appropriately trained radiologist. A wedge resection was performed with 1 cm parenchymal margins using combination of monopolar scissors, bipolar maryland, and LigaSure device. The specimen was removed in a specimen pouch through the pfannenstiel incision. After ensuring hemostasis the robot was undocked, with a total liver resection time, including docking, of approximately 180 min.

Given the proximity of our distal margin to the anal verge (approximately 5 cm), our tumor‐specific TME, and the potential morbidity with an anastomotic leak, it was decided to undergo proximal diversion. A loop ileostomy was fashioned in the usual manner using laparoscopic assistance. The patient was subsequently awoken from anesthesia and extubated. Blood loss was estimated to be approximately 300 cc. Total operative time was approximately 390 min. The patient was discharged on postoperative day six, tolerating a full diet. The final pathology showed a moderately differentiated adenocarcinoma with 2/36 positive nodes and clear microscopic margins at both the LAR and liver specimens. No lymph‐vascular or perineural invasion was reported. Final staging was determined to be pT3N1bM1a.

## Outcome and Follow‐up

Upon completion of pseudo‐adjuvant systemic therapy, the patient underwent a hypaque enema prior to reversal of his ileostomy. This revealed no evidence of an anastomotic leak; a subsequent flexible sigmoidoscopy, however, revealed a low‐grade stricture at the colorectal anastomosis requiring balloon dilatation. The patient went on to receive an uncomplicated reversal of his ileostomy followed by scheduled outpatient surveillance. At the 20‐month mark, the patient demonstrated no clinical or radiographic signs of recurrence. They were tolerating a normal diet and slowly regaining regularity in bowel movements, without any evidence of LAR syndrome on clinical assessment.

## Discussion

Interest in expanding the da Vinci's^®^ application to colorectal and liver procedures has been driven in part by the advantages it has over conventional laparoscopy, including greater range of articulation, tremor reduction, as well as improved depth perception and ergonomics. As demonstrated in this case, robot‐assisted simultaneous resection of both CRC and CRLM is feasible and safe.

Total mesorectal excision, the gold standard procedure for rectal cancer, requires meticulous and precise dissection under direct vision. The complex anatomy of the pelvis, restricted space and visibility, together with diminished dexterity with laparoscopic instruments makes laparoscopic TME one of the most challenging of minimal access procedures. A robotic approach may offer a solution to some of these challenges 15, 16. Systematic reviews and meta‐analyses comparing robotic‐assisted laparoscopic surgery (RALS) in colorectal cancer with conventional laparoscopic surgery (CLS) generally demonstrate a longer operative time when using robotic assistance 15, 16, 17. Examining randomized controlled trials (RCT) comparing the two approaches reveals that the difference in operative time is not significant 18. Studies have also shown that colorectal RALS has less estimated blood loss (EBL) compared to colorectal CLS 15, 16, 17, 18. Comparing other markers, there are no differences between either approach with respect to either days to soft diet or return of bowel function 15, 16, 17, 18. Analyzing only RCTs, however, suggests that patients return to bowel function sooner with RALS than with CLS 18. Comparing length of stay (LOS) with respect to both approaches yields contentious results. Some meta‐analyses report no difference in LOS 17, while others favor RALS as having a slightly shorter LOS 16. Rates of conversion to open surgery have been reported to be between 2% and 9.5% with RALS 15, 16, 17, 18, 19. One pooled analysis reported conversion rates twice as high with RALS 17 while another reported greater rate of conversion with CLS, with magnified differences in the rectal cancer population 16.

Minimally invasive approaches in liver surgery have the potential to offer many of the same benefits as in colorectal surgery. There have been two published systematic reviews examining RALS for liver resections. Mean operative times ranged from 200 to 507 min between both reviews. The mean LOS ranged from 5.5 to 11.7 days and the conversion rates were between 4.6% and 6.6%. No perioperative mortalities were reported in any of the studies in either review 20, 21. Of the studies comparing RALR with conventional laparoscopic liver resection (CLLR), blood loss, resection margins, LOS, and morbidity were similar with the two approaches 11, 12, 13, 14. Conversion rate was reported in one study and was found to not be different between CLLR and RALR 14.

A minimally invasive approach for primary CRC and liver metastasis allows for a complex operation with faster recovery and better outcomes compared to a conventional open approach. A recent systematic review by Lupinacchi et al. identified 14 studies involving laparoscopic simultaneous resections of both the primary colorectal lesion and the CLRM. There was no conversion to open resection, and mortality was null. Estimated blood loss varied between 10 and 650 mL and was not related to the type of liver or colorectal resection preformed. Length of hospital stay ranged from 4 to 54 days with an average of 9 days 22. However, utility and feasibility of robotic‐assisted simultaneous resection for CRC and CRLM have not been clearly demonstrated in the past. Interest in expanding the da Vinci's^®^ application to colorectal and procedures has been driven in part by the advantages it has over conventional laparoscopy, including greater range of articulation, tremor reduction, as well as improved depth perception and ergonomics.

Given the potential benefits of the da Vinci system over laparoscopic surgery at the operator level, its application to complex simultaneous resections could potentially be advantageous. There are case reports of combined colorectal and prostate or uterine procedures 23, 24]. However, there are far fewer reported cases of full robot‐assistance in resection of both CRC and CRLM. Our literature search revealed only two other studies that reported such procedures (Table 1). The first involved a simultaneous da Vinci^®^‐assisted left lateral sectionectomy and low anterior resection for a rectosigmoid primary with an associated 1.5 cm mass on segment 3 of the liver 25. The second involved a one‐stage da Vinci^®^‐assisted resection of a primary rectal cancer 13 cm from the anal verge with liver and lung metastases 26. Our present case, demonstrates blood loss and operative times comparable to these aforementioned studies. Similarly, we demonstrated equivalent oncologic and 30‐day outcomes with a shorter length of stay. Our length of stay of 6 days using the robotic approach was lower than the average of 9 days demonstrated by Lupinacchi et al., in a systemic review of combined laparoscopic liver and rectal resection 22. Our total operative time of 390 min is in keeping with the two prior reports of combined robotic resection (360–480 min) 25, 26. Furthermore our operative duration is also concordant with a recent study of 142 cases of combined laparoscopic combined colorectal and liver resections demonstrating a median operative time of 360 min 27. This case demonstrates that using the DaVinci‐S model, simultaneous resection can be conducted in a time conscious manner while also providing the additional benefits of the robotic platform for difficult to reach laparoscopic lesions of the liver, including those high over the dome of the diaphragm. Newer robotic platforms such as the DaVinci Xi have the potential to further reduce operative times by reducing docking times secondary to their boom mounted arms.

**Table 1:** Literature review summary of fully robot‐assisted resection of CRC and CRLM

Study	No. of Patients	No. of Patients Liver RALS	No. of Patients Colorectal RALS	EBL	Operative Time	LOS	30‐day Morbidity	Oncologic Margins
Choi 2008	1	1	1	700	360	13	None	Free
Xu 2015	1	1	1	600	480	7	None	Free
Current case	1	1	1	300	390	6	None	Free

CRC, Colorectal cancer; CRLM, colorectal liver metastasis; EBL, estimated blood loss; LOS, length of stay; RALS, robotic‐assisted laparoscopic surgery.

We believe this case highlights several important points. Firstly, application of the robot‐assisted daVinci system is feasible in the hands of well‐trained surgeons. Secondly, this report demonstrates that highly complex robotic‐assisted surgery can be conducted safely with regard to the intraoperative and postoperative management of patients with CRC and CRLM. Finally, this case also demonstrates that a robotic‐assisted simultaneous resection for CRC and CRLM has the potential for demonstrating good short‐ and long‐term oncologic outcomes with the potential for shorter length of stay. Further research involving larger cohort of patients is needed to confirm the efficacy of simultaneous robot‐assisted surgery with regard to both short and long‐term metrics.

## Consent

Written informed consent was obtained from the patient for publication of this Case report and any accompanying images. A copy of the written consent is available for review by the Editor‐in‐Chief of this journal.

## Authorship

SS, JR, AA, and DH: participated in the acquisition and analysis of data, literature review, and writing of the manuscript. SC and FAQ: were involved in the medical care of the patient, drafting of the manuscript, and acquisition and analysis of data. All authors approved the final version of the manuscript.

## Conflict of Interest

None declared.
